# Supplementary material for: Nontargeted Metabolomics by High-Resolution Mass Spectrometry to Study the In Vitro Metabolism of a Dual Inverse Agonist of Estrogen-Related Receptors β and γ, DN203368
Source: Pharmaceutics. 2021 May 31;13(6):776. doi: 10.3390/pharmaceutics13060776 (PMC8230175; doi:10.3390/pharmaceutics13060776)
Supplement: Supplementary file 1 [file pharmaceutics-13-00776-s001.zip › pharmaceutics-1204241-SI.pdf]

pharmaceutics

MDPI

Supplementary Materials: Nontargeted Metabolomics by High-Resolution Mass Spectrometry to Study the In Vitro Metabolism of a Dual Inverse Agonist of Estrogen-Related Receptors β and γ, DN203368

Sin-Eun Kim, Seung-Bae Ji, Euihyeon Kim, Minseon Jeong, Jina Kim, Gyung-Min Lee, Hyung-Ju Seo, Subin Bae, Yeojin Jeong, Sangkyu Lee, Sunghwan Kim, Taeho Lee, Sung Jin Cho and Kwang-Hyeon Liu

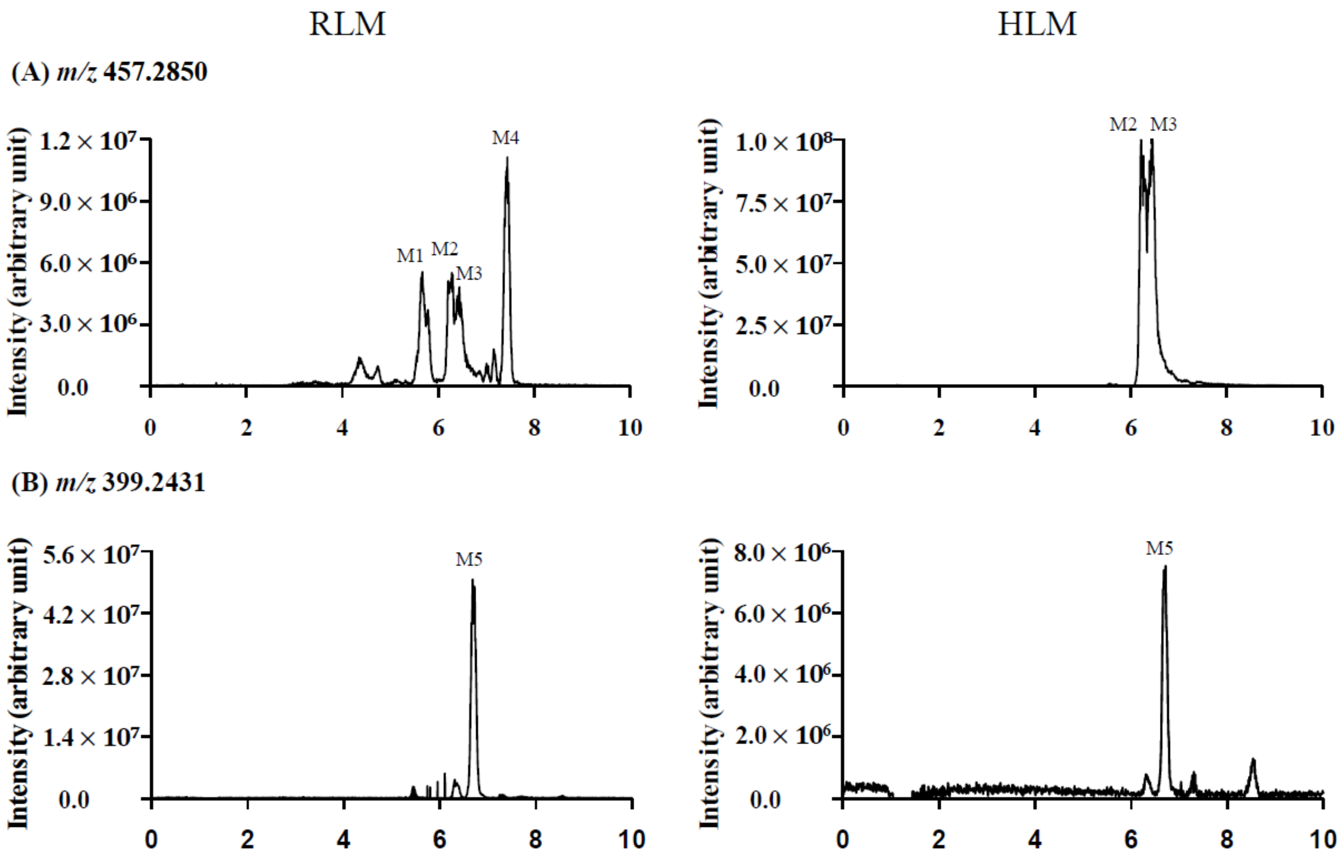

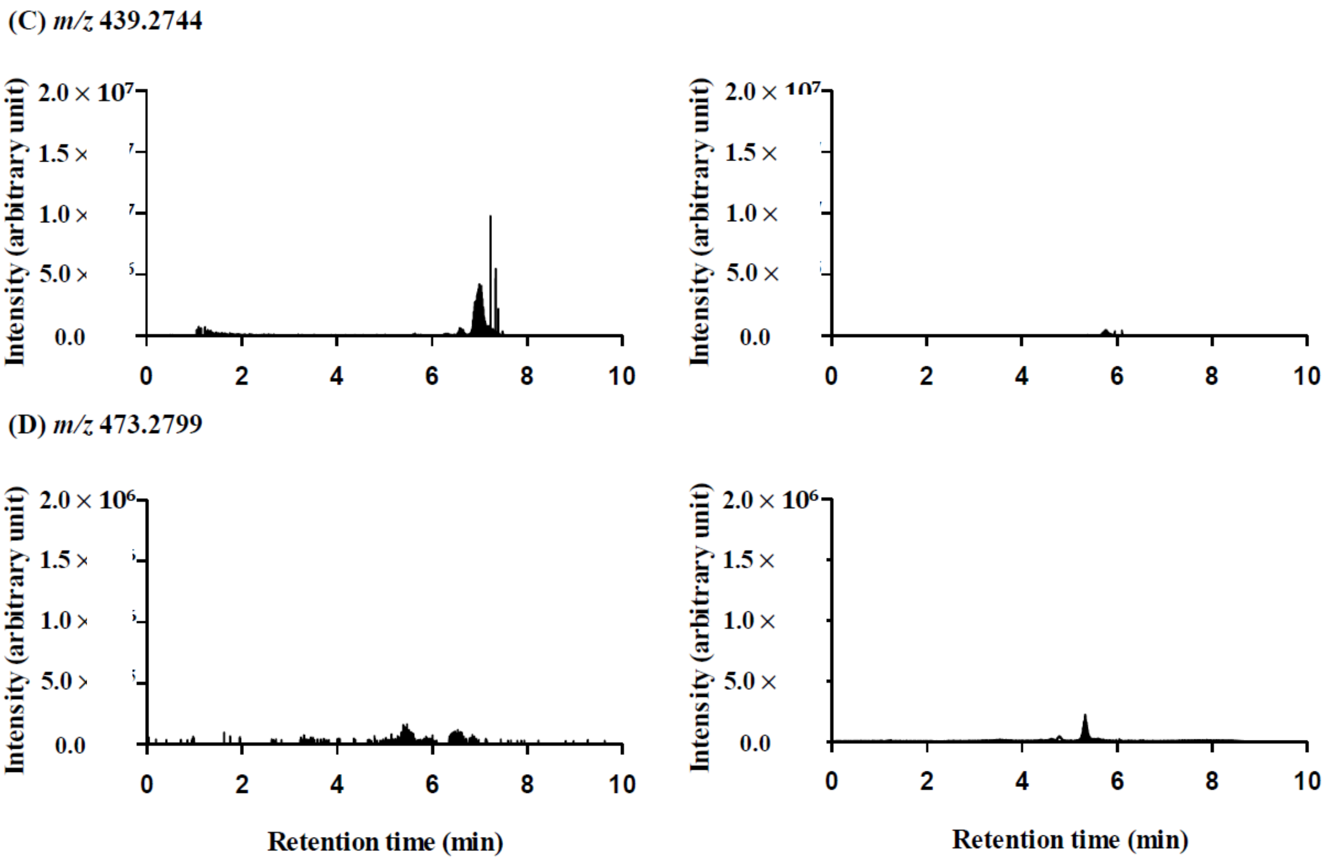

Figure S1. Extracted ion chromatograms (EIC) of DN203368 metabolites obtained from rat and human liver microsomal (RLM, HLM) incubates with DN203368 in the presence of NADPH generating system.

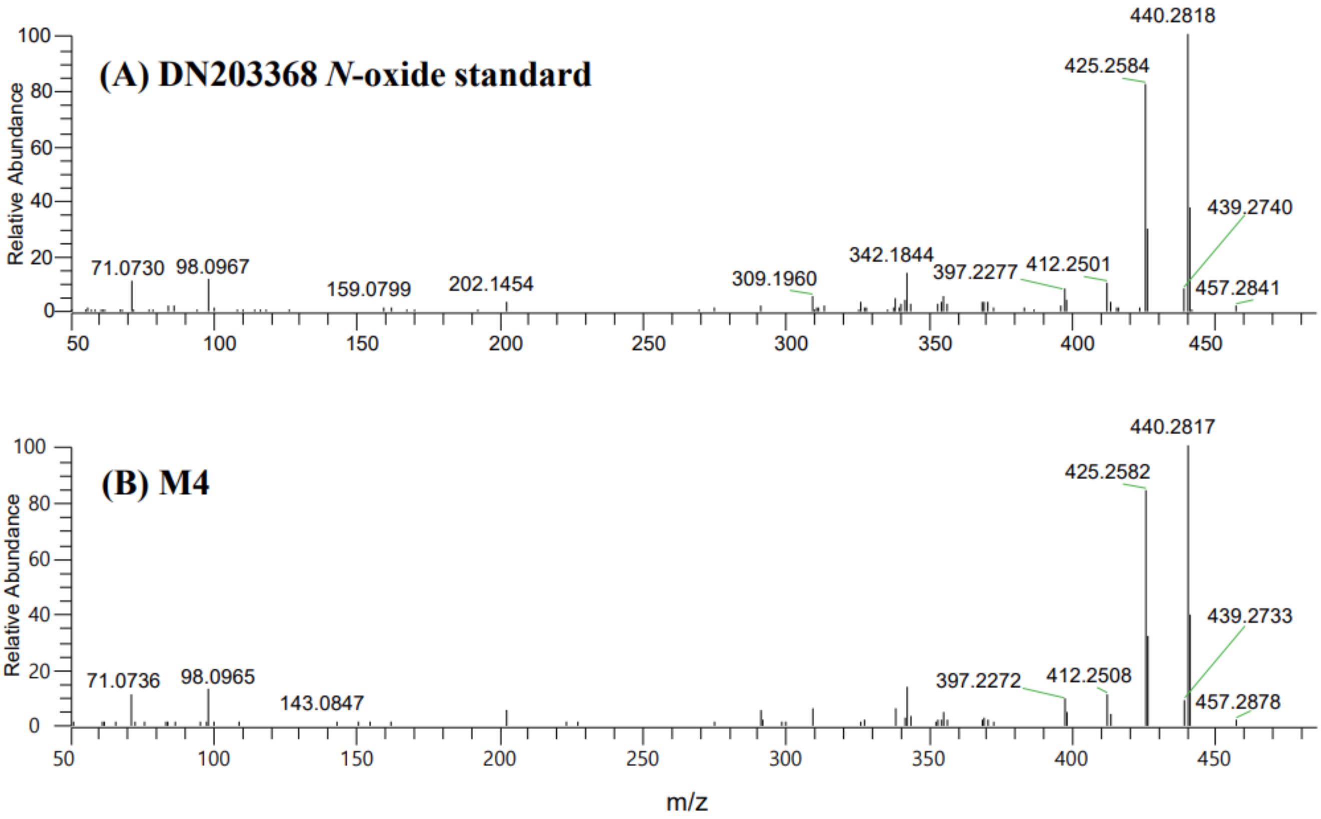

Figure S2. Product ion mass (MS/MS) spectra of DN203368 *N*-oxide standard (**A**) and metabolite M4 (**B**).
